# A pharmaceutical monitoring system to assess the quality of antituberculosis drug products used in Mauritania

**DOI:** 10.1371/journal.pone.0282023

**Published:** 2023-03-16

**Authors:** Amor R. Cáceres-Pérez, Mohamed B. El Kory, Javier Suárez-González, Mabel Soriano, M. Magdalena Echezarreta, Ana Santoveña-Estévez, José B. Fariña

**Affiliations:** 1 Facultad de Farmacia, Universidad de La Laguna, San Cristóbal de La Laguna, Spain; 2 Programa de Doctorado Ciencias Médicas y Farmacéuticas, Desarrollo y Calidad de Vida, Universidad de La Laguna, San Cristóbal de La Laguna, Spain; 3 Instituto Universitario de Enfermedades Tropicales y Salud Pública de Canarias, Universidad de La Laguna, San Cristóbal de La Laguna, Spain; 4 Laboratoire National de Contrôle de la Qualité des Médicaments, Nouakchott, Islamic Republic of Mauritania; University of Turin, ITALY

## Abstract

The quality of drug products may be affected from manufacture to dispensing, particularly at high temperature and humidity as in Mauritania. This country is not included in the World Health Organization reports on poor quality products due to the lack of a qualified laboratory and monitoring system. Ensuring the quality of medicine is even more relevant in the case of diseases such as Tuberculosis, due to its high prevalence, complex treatment and continuous bacterial resistance. The aim was to develop a monitoring system to assess the quality of antituberculosis drugs products, by the substandard detection based on European and United States Pharmacopeial recommendations regarding quality control. In addition to studying the influence of accelerated storage conditions (40 ± 2°C/75 ± 5% relative humidity) on their qualities and comparing the dissolution profiles to contrast the quality. 18 antituberculosis drug products were taken from Europe and Mauritania, and quality was studied through visual inspection and according to the compliance of the mass uniformity, uniformity of dosage units, dissolution, disintegration and friability pharmacopeial tests. Furthermore, a dissolution profile comparison was carried out to examine quality. A stability study was conducted to assess the influence of climatic conditions on the content and the dissolved amount of the active pharmaceutical ingredients, which were determined by an ultra-performance liquid chromatography system. As result, 69.3% of 13 Mauritanian formulations had a substandard quality mainly due to non-compliance with the test for friability or content uniformity of these medicines. All European drug products complied with pharmacopeia specifications. In addition, storage conditions affected the dissolution rate of ethambutol and the uniformity of the 4 antituberculosis combination drug products.

## Introduction

Market globalisation and the increased complexity of the drug products supply chain has led to the inclusion of poor-quality medical products within the pharmaceutical market. This global issue is even more serious in low and middle income countries (LMIC) because of the lack of resources to detect substandard drug products [1, 2].

These types of medical products include substandard, falsified and unregistered drug products. Substandard drug products, also referred to as out of specification, are authorized drug products that fail to meet either their quality standards or their specifications, or both. Falsified drug products whose composition, identity or manufacturing origin are misrepresented deliberately. Unregistered drug products are not authorised to be commercialized or approval is under evaluation [3].

These falsified and substandard medical products represent a loss of confidence in the health system, as they pose a health risk by prolonging illness, promoting drug-resistant infections, or causing deaths because of the product itself or the lack of treatment. For example, during the manufacture of certain drug products like ranitidine, metformin or angiotensin II receptor blockers, the formation of impurities such as nitrosamines must be controlled to avoid adverse health effects [4].

In this sense, in 2013 the World Health Organisation (WHO) launched a Global Surveillance and Monitoring System (GSMS) to improve the quantity, quality and analysis of accurate data to prevent the presence of these products, detect them and respond quickly and effectively to their removal from the market. From 2013 to 2017 GSMS received 1500 reports, 42% of them from Africa. A large proportion of these reports were found in countries such as Mali, Nigeria, Angola, Democratic Republic of The Congo or Cameron. According to GSMS the products detected as substandard and falsified were above all malaria medicines, antibiotics, anaesthetics/painkillers and lifestyle products with 19.6, 16.9, 8.5 and 8.5 of percentage of all products reported to database [1]. However, it is difficult to know the current absolute number of the substandard and falsified medical products because WHO-accredited reference laboratories are needed to detect and report the presence of substandard medical products [3].

Furthermore, there are other platforms such as the infectious diseases data observatory (IDDO), which has developed a drug quality map based on data from different studies published since 1985. This global map indicates the samples analysed specifying the sampling site and its quality results. Moreover, a colour system has been established to visually identify the failure quality rate of these studies which show that the countries with a major failure rate are India and Kazakhstan in Asia and Chad, Kenya, Nigeria, and Sudan in Southern Africa. The IDDO platform has had 761 reports over the last 10 years, 34.3% were from antibiotics and 13.3% from antimalarials. As for antituberculosis (antiTB) drug products a mere 13 studies were reported during that same period, all of which were from African, Asian and American [5].

The presence of platforms such as IDDO and the publication of reports by international organizations about the presence of substandard and falsified medicines is extremely important as an indication of the quality of the pharmaceutical market in a specific region. Thus, this information, will encourage countries to develop a national action plan to prevent this state of affairs, implant track and trace systems, ensure supply chain integrity and implement reporting systems which will protect public health and prevent recurrence.

In addition, the quality of medical products may be affected in each step of its lifecycle: manufacture, shipping and storage, as well as climatic conditions which may not ensure the quality of many drug products. This is of particular interest in countries in climate zones type IV, where temperature and relative humidity (RH) are high. Therefore, an appropriate sampling strategy has to be followed to collect a large number of units of each drug product, sampled from different points of the supply-chain, to perform all pharmacopoeial tests.

Some studies indicated correct quality of the drug products tested although these studies did not follow the recommendations of European or United States Pharmacopeia, because they did not calculate the acceptance value (AV) for uniformity of dosage units, or used an incorrect number of units [6, 7]. In this regard, it is very common to find in African research groups studies conducted with the minilab, which is a semi-quantitative technique that is not suitable for the detection of substandard drug products [8].

The prevalence of substandard and falsified antiTB drug products in LMIC poses health issues that have been exacerbated, by the prevalence, the complexity of treatment and increasingly resistant diseases due partly to the commercialization of these kinds of products [9]. For example, TB killed in 2019 1.4 million of people and is highly relevant and a considerable burden in sub-Saharan Africa; Senegal, Somalia or Kenya with 220, 117, 259 and 260 cases per 100.000 people respectively. Treatment is complex and divided in 2 different phases. An intensive phase with rifampicin, isoniazid, pyrazinamide and ethambutol that is administered for 2 months, followed by the maintenance phase for 4 months using rifampicin and isoniazid [10, 11]. In addition, in certain African countries like Islamic Republic of Mauritania (hereafter referred as Mauritania), the prevalence of Multidrug-resistant TB (MDR-TB) is around 30–49% and requires the inclusion of other active pharmaceutical ingredients (API) such levofloxacin for treatment. The quality assurance of the second line treatment is essential to cure the disease and prevent new resistances, especially in these countries [12]. For antiTB drug products which contain isoniazid, the most important impurity is hydrazine which has a potential carcinogenic risk and a limit value of 39 μg/ml as ICH indicated [13]. Furthermore, degradation occurs by the combination of rifampicin and isoniazid stored under accelerated conditions [14, 15].

In order to reduce the presence of substandard and falsified products in pharmaceutical markets, the WHO has recommended certain steps which are based on the prevention, detection and a rapid response to these products. Training and the support of a network of nationally designated focal points within national and regional regulatory agencies is fundamental. These act as a channel of communication, develop tools and systems that countries can adapt to make reporting of suspected products easier and more efficiently, develop a global database of reports, prequalification of manufactures and laboratories among other endeavours.

It is therefore clear that quality assurance systems for drug products are needed in the light of these findings. Thus, a research project called ISACAM (Instauración de un Sistema para el Aseguramiento de la Calidad de Medicamentos utilizados en el tratamiento del SIDA y enfermedades tropicales descuidadas, from Spanish) aims to establish a quality assurance system for drug products used to treat TB, malaria and HIV/AIDS [16]. Due to the fact that there are no reports of quality of drug products marketed in Mauritania, this country was selected to carry out the project, in collaboration with the Laboratoire National de Contrôle de la Qualité des Médicaments (LNCQM), a department of the Ministry of Health of Mauritania. As part of quality assurance, quality control is essential and must be carried out by certified and qualified laboratories to detect substandard drug products. Hence, ISACAM project will also contribute to promote the prequalification of LNCQM as a WHO reference laboratory for the detection of falsified and substandard medical products.

The present work is part of the ISACAM research project with the aim to assess the quality of the main antiTB drug products used in the Mauritanian health system, by developing a monitoring system to detect substandard drug products through the performance of pharmacopeial tests, as well as contrasting the quality of medicines with European equivalents if available. The study was completed by monitoring the stability of the selected antiTB drug products after storage in a climatic chamber, simulating the harsh climatic conditions of Mauritania, to find out whether the storage conditions are relevant for the emergence of substandard medicine.

## Materials and methods

### Materials

The following materials were used: Levofloxacin (**≥**97.3%, Mylan Laboratories Limited®, Spain), Pyrazinamide (**≥**99.0%, Sigma-aldrich®, China), Rifampicin (**≥**97.0%, Sigma-aldrich®, China), Isoniazid (**≥**100.2%, Acofarma®, Spain), Ethambutol (**≥**99.0%, Sigma-aldrich®, USA), Hydrazine monohydrate (**≥** 98%, Sigma-aldrich®, Germany), hydrochloric acid solution 1M (Fluka®, Germany), formic acid (Fluka®, Switzerland), sodium chloride (Sigma-aldrich®, Denmark), di-potassium hydrogen phosphate (Merck®, Germany), potassium dihydrogen phosphate (Supelco®, Germany), di-sodium hydrogen phosphate anhydrous (Panreac®, USA), acetonitrile (Sigma-aldrich®, Germany), triethylamine (Sigma-aldrich®, Belgium) and benzaldehyde (Sigma-aldrich®, Belgium). Purified water was obtained from a water purification system (Puranity TU 12, VWR, Radnor, PA). APIs were analysed before being used and compared with technical sheet provided by the manufacture.

### Sampling

Formulations were selected to be analysed based on their use as first line or MDR treatment for TB in the Mauritanian health system. They were sampled in Spain and Mauritania, in Nouakchott, at different geolocated sampling sites. The selection of the formulations, as well as the sampling site, depended on the availability of TB medicines, due to the lack of these in the country. Specifically in Mauritania, samples were taken from distribution or health centres like centres of diagnostic and treatment (CDT), Programme National de Lutte contre Tuberculosis et Paludisme (PNLTP, from French), Hôpital Polyclinique (HP, from French) or Centre de santé de Araffat IBN Sina (CS IBN, from French).

An identification sheet was filled *in situ* for each drug product with relevant information related to packaging (batch number, number of units, expiration date, manufacturer laboratory and packaging conditions), storage place (establishment type and name, and its storage conditions), person responsible of sampling and date. After collecting the drug products, they were placed in an isothermal bag and transported to the laboratory for quality control. Upon arrival at the laboratory, the drug products were stored at 5°C and 11% RH and analysed before their expiration dates.

### Analytical methods validation

To proceed with the analysis of the APIs of the selected drug products, an Acquity Ultra Performance Liquid Chromatography (UPLC)® H-Class System (Waters, Milford, MA, USA) was used. The data acquisition software was Astra 6.0.1. (Chromatographic Manager, Waters Corporation).

One analytical method was validated for each standard product according to International Conference Harmonization ICHQ2(R1) guideline, ensuring its linearity, precision, accuracy, and specificity [17]. An analysis of origins of variations (ANOVA) was done using Excel® (Microsoft corporation, USA) to confirm the linearity for all API studied through the rejection of the null hypothesis (α = 0.05). The accuracy, expressed as recovery percentage, was determined by analysis of a known concentration of standard (n = 9) and precision, expressed as repeatability, was calculated by testing the same sample six times. Limit of detection (LOD) and quantitation (LOQ) were also determined.

Table 1 shows a summary of the UPLC method conditions used for all products. Levofloxacin method was adapted for UPLC from a previously published article developed for High Performance Liquid Chromatography [18]. Rifampicin, isoniazid, pyrazinamide and hydrazine methods were previously validated and published by the investigation group [19, 20]. For the analysis of these products a reversed phase C18 column (X-Select® CSH C18 [2.1 × 75 mm; 2.5 μm] (Waters, USA)) was used as stationary phase. For the analysis of ethambutol a method adapted from United States Pharmacopeia (USP) was used employing a nitrile group column (XSelect HSS CN [2.1 x 100 mm, 2.5 μm] (Waters, USA)) [21]. The injection volume was 10 μl for all API and 1 μl for hydrazine.

**Table 1:** Conditions of UPLC methods.

Products	Mobile phase		Flow rate (ml/min)	Wavelength (nm)	Concentration range (μg/ml)
	A	B			
LVFX	ACN (20%)	FA [0.05% (v/v)] in water (80%)	0.3	295	1–5 (n = 32)
R	ACN (38%)	*KH*_2_*PO*_4_ buffer, 30 mM pH = 3.7 (62%)	0.5	254	10–27 (n = 30)
H	ACN (2%)	*KH*_2_*PO*_4_ buffer, 30 mM pH = 3.7 (98%)			10–22.5 (n = 30)
Z					10–22.5 (n = 30)
E	ACN (50%)	TEA [0.1% (v/v)] in water	0.8	200	30–750 (n = 32)
Hydrazine	ACN (50%)	Water (50%)	0.4	300	3.1–15.4* (n = 42)

LVFX: Levofloxacin; R: Rifampicin; H: Isoniazid; Z: Pyrazinamide; E: Ethambutol; ACN: Acetonitrile; FA: Formic acid; TEA: Triethylamine.

*This concentration is expressed in ng/ml.

0.5 mg/ml levofloxacin standard solution was prepared in hydrochloric acid 0.1 N. In the case of ethambutol, 15 mg were dissolved in 10 ml of purified water. Then, these solutions were diluted to obtain concentration between 1–5 μg/ml and 30–750 μg/ml for levofloxacin and ethambutol respectively. All samples and solvents were filtered with 0.2 μm pore-size filters (Millipore, USA).

### Sample preparations

Preliminary API extraction yields from drugs were performed by triplicate to confirm that the sample preparation procedure was satisfactory. Then, every solid pharmaceutical form was powdered, before being analysed, and the following procedure was carried out:

- A levofloxacin film-coated tablet (FCT) was transferred to a 1 L flask, fulfil 75% of the volume with 0.1 N HCl to be dissolved. Then, volume was completed and stirred at 750 rpm for 10 minutes, getting a concentration of 0.5 mg/ml.
- An uncoated tablet (UT) of pyrazinamide was sonicated in a 250 ml flask with purified water for 10 minutes for proper dissolution, 1.6 mg/ml.
- An ethambutol UT was taken and transferred to 250 ml flask, then purified water was added and stirred until complete dissolution, 1.6 mg/ml.
- An isoniazid UT was dissolved in 375 ml of purified water with magnetic stirring at 700 rpm for 30 minutes in a 500 ml flask. Finally, the flask was filled up, 0.6 mg/ml.
- Rifampicin and isoniazid were extracted from fixed dose FCT, by dissolving each tablet in 75 ml of methanol. Then, purified water was added up to 250 ml flask to complete the dissolution, 0.6 and 0.3 mg/ml. The same procedure was followed for rifampicin, isoniazid, pyrazinamide and ethambutol tablets obtaining concentrations of 0.6, 0.3, 1.6 and 1.1 mg/ml respectively.
- A rifampicin, isoniazid and pyrazinamide fixed dose FCT was transferred to 250 ml flask, dissolved in 75 ml of methanol, filled up to 75% of the total capacity with purified water and sonicated for 20 minutes. Finally, the volume was completed with purified water till 0.5 mg/ml of rifampicin, 0.2 mg/ml of isoniazid and 1.2 mg/ml of pyrazinamide.

Finally, each solution was diluted in order to obtain concentrations between the concentration ranges according to analytical method, Table 1.

### Quality control of pharmaceutical dosage forms

The quality control of oral dosage forms included a visual inspection and the tests recommended in European Pharmacopeia (Eur. Ph.) and USP for all drug products studied [22, 23].

#### Visual inspection

Primary and secondary packaging were checked for imperfections, colour changes and lack of relevant information according to regulatory agencies.

#### Mass uniformity test

20 tablets were used to establish the mean mass and standard deviation of the measures according to pharmacopeial recommendations [24].

#### Uniformity of dosage units test

Mass variation or content uniformity (CU) tests were carried out according to the Eur. Ph. [25]. Calculating the AV is necessary to ensure the uniformity between units and its pharmacopeial limit is set at 15. Samples were dissolved following sample preparation procedures and diluted to a concentration that could be quantified by UPLC.

#### Dissolution test

Dissolution tests were performed following USP recommendations for each drug product, see Table 2, using a dissolution tester DT 1410 (Erweka, Germany) [21, 26–31]. Finally, samples were filtered (3 mm 46 x 57 cm Whatman, UK), diluted and analysed by UPLC.

**Table 2:** Conditions for each dissolution test.

API dose and pharmaceutical form	Apparatus	Medium	Rotation speed (rpm)		Time (minutes)	Q (%)
LVFX tablets	Paddle	HCl 0.1 N	75	30		LVFX: 80
Z tablets	Paddle	Water	50	45		Z: 75
E tablets	Basket	Water	100	45		E: 75
H tablets	Basket	HCl 0.01N	100	45		H: 80
R and H capsules*	Basket	HCl 0.1 N	100	45		R: 75 H: 80
R, H and Z tablets	Basket	Simulated gastric fluid TS, without pepsin	100	30		R and H: 80 Z: 75
R, H, Z and E tablets	Paddle	*Na*_2_*HPO*_4_ buffer, 10 mM (pH = 6.8)	100	45		R, H, Z and E: 75

API: Active Pharmaceutical Ingredient. LVFX: Levofloxacin; R: Rifampicin; H: Isoniazid; Z: Pyrazinamide; E: Ethambutol; HCl: hydrochloric acid.

*This condition corresponds to rifampicin and isoniazid capsules.

#### Disintegration test

Disintegration time of 6 tablets of each drug product was determined using a ZT disintegrator tester (Erweka, Germany) following Eur. Ph. recommendations [32].

#### Friability test

Friability test was done following the recommendations of Eur. Ph. monograph using a Tablet Friability/Abrasion Tester TAR Series (Erweka, Germany) only for UT [33].

### Dissolution profile comparison

A dissolution profile comparison was carried out, calculating the difference factor (*f1*, Eq 1) and similarity factor (*f2*, Eq 2), according to Food and Drugs Administration (FDA) and European Medicine Agency (EMA) recommendations, if a reference drug product was available in the European market for a Mauritanian formulation of the same API [34, 35].


$$f_{1}=\left[\frac{\sum R_{t}-T_{t}}{\sum R_{t}}\right]\cdot 100$$ Eq 1

Where R_t_ is the dissolution value of the reference formulation at time t and T_t_ is the dissolution value of the test formulation at time t.


$$f_{2}=50\cdot log\left[\frac{100}{\sqrt{1+\frac{\sum_{t=1}^{t=n}{\left[\bar{R}(t)-\bar{T}(t)\right]}^{2}}{n}}}\right]\cdot 100$$ Eq 2

Where n is the number of time points, R(T) is the mean percent reference drug dissolved at time t after initiation of the study; T (t) is the mean percent test drug dissolved at time t after initiation of the study.

In order to establish the similarity between dissolution profiles, *f1* must be up to 15 and *f2* between 50 and 100. 12 tablets of each drug product were analysed to describe their dissolution profile. Samples of 5 ml were withdrawn, and replaced with new medium at 5, 10, 15, 20, 30 and 40 minutes, diluted and finally analysed by UPLC.

Different statistical test could also be performed according to FDA and EMA as the model independent multivariate confidence region procedure or the model dependent approach. This was performed in cases where coefficient of variation within formulation is more than 15% or when a more suitable model was required respectively.

### Stability study

For stability study, only drug products which complied with previous assays of uniformity of dosage units and dissolution tests at initial time, were stored under accelerated conditions (40 ± 2°C/75 ± 5% RH) in a climatic chamber (ICH 110L, Memmert) [36]. The assessment of the impact of these conditions was carried out by performing of uniformity of dosage unit and dissolution test, at 3 and 6 months. The detection of hydrazine content was also performed for 3 tablets of drug products stored when applicable.

## Results and discussion

### Sampling

Table 3 shows the 18 collected drug products as well as their sampling sites, manufacturer countries and the codification employed to identify them for quality control and stability studies. All drug products were commercialized in blister.

**Table 3:** List of antituberculosis drug products sampled.

API dose and pharmaceutical form	Drug products	Batch number	Manufacture date	Expiry date	Sampling site	Manufacturer country	Code
LVFX: 500 mg *FCT*	*Levofloxacino Cinfa ®*	BP3234	09/2018	08/2022	Spain—ML	Spain	LVFX-ML
	*Tavanic ®*	7TP3C	12/2017	12/2020	Spain—DC	Spain	LVFX-DC
	*Amesol ®*	E2M103	12/2018	12/2023	Mauritania—PNLTP	Cyprus	LVFX-PNLTP
P: 400 mg *UT*	*Pyrazinamide 400 mg*	16TPF030F	10/2016	09/2020	Mauritania—PNLTP	India	Z-PNLTP
		NPB919A	11/2019	09/2023	Mauritania–CS IBN	India	Z-IBN
E: 400 mg *FCT*	*Myambutol ®*	19001T	10/2018	10/2024	Spain—DC	Spain	E-DC
E: 400 mg *UT*	*Ethambutol 400 mg*	EEZ923D	10/2019	09/2023	Mauritania—HP	India	E-HP
		EEZ643	11/2016	10/2020	Mauritania—PNLTP	India	E-PNLTP
I: 300 mg *UT*	*Isoniazid 300 mg*	NIB9588A	11/2019	10/2023	Mauritania—CS IBN	India	H-IBN
R: 150 mg I: 75 mg *FCT*	*Rifampicin 150 mg & Isoniazid 75 mg*	NRH9352A	11/2019	10/2022	Mauritania—CDT	India	RH-CDT
		NRH9352A	11/2019	10/2022	Mauritania—CS IBN	India	RH-IBN
		NRH9352A	11/2019	10/2022	Mauritania—HP	India	RH-HP
R: 120 mg I: 50 mg P: 300 mg *FCT*	*Rifater ®*	A7042	06/2017	06/2020	Spain—CP	Spain	RHZ-CP
R: 60 mg I: 30 mg P: 150 mg *FCT*	*Rifampicin 60 mg*, *Isoniazid 30 mg & Pyrazinamide 150 mg*	S46	07/2018	06/2021	Mauritania—CS IBN	India	RHZ-IBN
R: 150 mg I: 75 mg P: 400 mg E: 275 mg *FCT*	*Rimstar®*	LB1795	09/2020	09/2022	Spain—DC	Spain	RHZE-DC
	*Rifampicin 150 mg*, *Isoniazid 75 mg*, *Pyrazinamide 400 mg & Ethambutol Hydrochloride 275 mg*	NRG9208A	11/2019	10/2022	Mauritania—CDT	India	RHZE-CDT
		NRG20249A	10/2020	09/2023	Mauritania—CS IBN	India	RHZE-IBN
		NRG20249A	10/2020	09/2023	Mauritania—HP	India	RHZE-HP

API: Active Pharmaceutical Ingredient. LVFX: Levofloxacin; R: Rifampicin; I: Isoniazid; P: Pyrazinamide; E: Ethambutol. FCT: Film-coated tablet. UT: Uncoated tablet. ML: Manufacturer Laboratory. DC: Distribution company. PNLTP: Programme National de Lutte contre Tuberculosis et Paludisme. CS IBN: Centre de santé de Araffat IBN Sina. HP: Hôpital Polyclinique. CP: Community Pharmacy. CDT: Center of diagnostic and treatment

### Analytical methods validation

Previously, the methods to detect and quantify rifampicin, isoniazid, pyrazinamide and hydrazine were successfully validated, being linear, precises and accurate as can be found in previously published studies [19, 20].

ANOVA was done, confirming the method linearity for a significant level of 0.05 (α = 0.05), obtaining the following equation for levofloxacin: Area (μV*sec^-1^) = 160117 + 175080*C (μg/ml); r^2^ = 0.981. Coefficient variation (CV) of this method was 4.3%. It was precise (0.7%) and accurate (101.3%) because these values met with the limit values established, lower than 1% and between 97–103% for being precise and accurate, respectively. Its LOD and LOQ were 0.6 and 1.8 μg/ml respectively.

The linearity of ethambutol method was also confirmed and its linear regression equation was Area (μV*sec^-1^) = 1605.2*C (μg/ml); r^2^ = 0.998, with a CV of 2.9%. Method was precise (0.2%), accurate (99.6%) and its LOD and LOQ were 30.7 and 93.1 μg/ml respectively.

**Fig 1:**
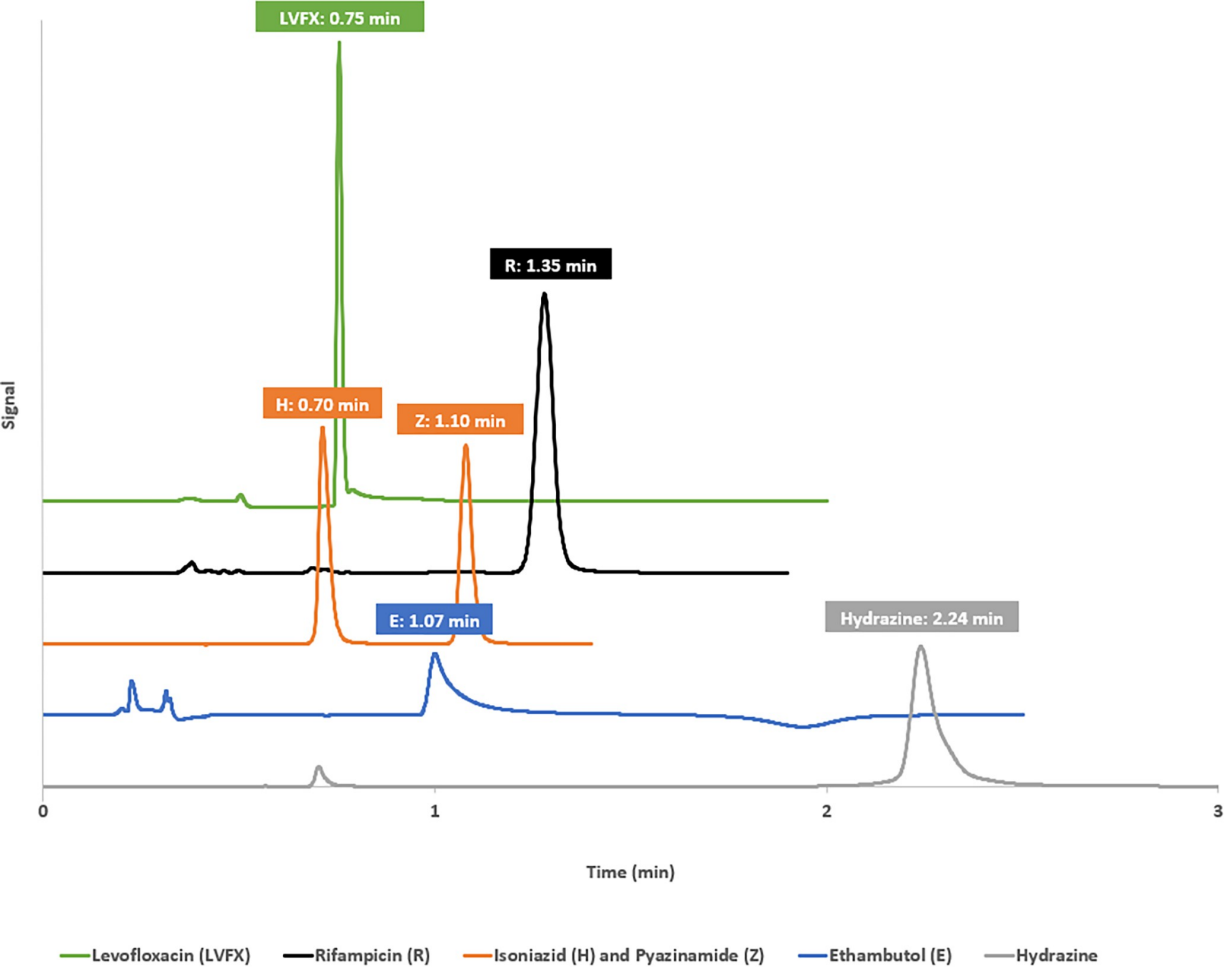
Chromatographs and retention times for levofloxacin (LVFX), rifampicin (R), isoniazid (I), pyrazinamide (Z), ethambutol (E) and hydrazine.

Chromatograms and retention times for each API analysed by the methods mentioned in Table 1 are shown in Fig 1.

### Quality control

All drug products studied complied with the mass uniformity test, as no individual units deviated more than 5% of mean mass. All tablets disintegrated in less than 15 minutes, as pharmacopoeia recommends. Furthermore, the content of hydrazine was quantified for 3 tablets of each drug product that contained isoniazid, without detecting this impurity in any sample. Results of uniformity of dosage units and dissolution tests are shown in Table 4, and they are discussed below.

**Table 4:** Results of uniformity of dosage units and dissolution tests after their collection.

		Quality control after collection					
		UDU test			Dissolution test		Pass/Fail
CODE	API	% DV	AV	n	% released	n	
LVFX-ML	LVFX	98.2 ± 0.5	1.5	10	86.3 ± 4.9	12	Pass
LVFX-DC		112.8 ± 0.5	12.4	10	84.0 ± 3.9	12	Pass
LVFX-PNLTP		107.4 ± 0.8	7.8	10	82.6 ± 2.8	12	Pass
Z-PNLTP	Z	95.2 ± 0.7	4.9	10	95.6 ± 1.1	6	Fail
Z-IBN		97.6 ± 1.9	5.3	10	100.4 ± 2.0	6	Fail
E-DC	E	95.2 ± 0.7	4.9	10	98.8 ± 1.2	6	Pass
E-PNLTP		98.6 ± 1.1	2.7	10	100.4 ± 4.4	6	Fail
E-HP		96.4 ± 1.5	4.6	10	97.5 ± 0.7	6	Fail
H-IBN	H	98.3 ± 1.8	4.3	10	102.5 ± 1.0	6	Fail
RH-CDT	R	101.2 ± 5.6	11.2	30	78.8 ± 7.1	12	Fail
	H	96.3 ± 6.4	15.0		97.4 ± 5.4		
RH-IBN	R	103.0 ± 5.5	12.5	30	92.8 ± 8.9	12	Fail
	H	104.3 ± 7.7	18.2		102.0 ± 3.0		
RH-HP	R	103.7 ± 8.7	19.7	30	90.4 ± 7.2	6	Fail
	H	105.2 ± 5.1	13.8		98.3 ± 6.3		
RHZ-CP	R	103.5 ± 1.9	6.5	10	88.1 ± 10.9	12	Pass
	H	100.9 ± 4.3	10.2		99.2 ± 2.9		
	Z	97.7 ± 2.0	5.6		99.2 ± 1.8		
RHZ-IBN	R	106.2 ± 5.2	15.1	30	29.1 ± 21.8	6	Fail
	H	103.5 ± 4.7	11.4		93.1 ± 6.9		
	Z	94.5 ± 1.8	7.5		99.0 ± 2.7		
RHZE-DC	R	102.4 ± 5.0	12.9	10	112.4 ± 2.8	6	Pass
	H	95.2 ± 2.7	9.9		106.1 ± 4.0		
	Z	98.1 ± 3.1	7.7		108.0 ± 2.7		
	E	92.6 ± 3.4	14.1		92.2 ± 1.4		
RHZE-HP	R	95.8 ± 2.9	9.6	10	95.0 ± 2.0	6	ND
	H	96.5 ± 2.4	7.6		101.0 ± 0.9		
	Z	103.3 ± 2.1	3.3		102.1 ± 1.8		
	E	91.4 ± 8.6	27.6		83.5 ± 2.4		
RHZE-IBN	R	96.3 ± 3.9	11.4	10	95.3 ± 2.4	6	Pass
	H	94.0 ± 1.5	8.2		99.3 ± 1.3		
	Z	98.2 ± 1.3	3.4		105.7 ± 0.7		
	E	97.0 ± 4.9	13.1		94.6 ± 0.9		
RHZE-CDT	R	99.4 ± 4.3	10.3	10	95.3 ± 3.2	6	Pass
	H	96.0 ± 3.2	10.1		102.3 ± 2.2		
	Z	99.1 ± 1.9	4.5		104.8 ± 3.0		
	E	98.6 ± 2.2	5.4		101.7 ± 2.4		

API: Active Pharmaceutical Ingredient. L: Levofloxacin; R: Rifampicin; I: Isoniazid; P: Pyrazinamide; E: Ethambutol. ML: Manufacturer Laboratory. DC: Distribution company. PNLTP: Programme National de Lutte contre Tuberculosis et Paludisme. CS IBN: Centre de santé de Araffat IBN Sina. HP: Hôpital Polyclinique. CP: Community Pharmacy. CDT: Center of diagnostic and treatment. UDU: Uniformity of dosage units. DV: Declared value. AV: Acceptance value. n: Number of tablets tested. ND: No determined.

#### Levofloxacin tablets

No defects were found in the packaging of all sampled levofloxacin formulations, 2 from Spain and 1 from Mauritania. The AV of all drug products was lower than the limit for 10 units of each drug complying with uniformity of dosage units test. Furthermore, the mean API content released from 12 tablets above 80% for each formulation, complying with dissolution test. However, in other African countries substandard drug products manufactured in Asian were found. These did not contain the amount of levofloxacin labelled, being below 90% of declared content. In addition, drug release was considered a problem in many cases [37–41]. For example, Zabala et al. (2022) categorized as substandard 29.8% of drug products sampled due to dissolution failure [42]. In this case, as the majority of samples taken from health Mauritanian system were from Indian, except levofloxacin drug product, presumably there could also be a substandard levofloxacin.

#### Pyrazinamide tablets

The 2 batches sampled at PNLTP and CS IBN were manufactured by the same laboratory and their packaging was satisfactory. Pyrazinamide content and their AV agreed with pharmacopeia limit, and the amount of this API released during dissolution test was above 80% for both drug products. Nevertheless, tablets from both batches broke during the friability test. Soremekun RO et al (2014) studied the friability of various drug products and found that pyrazinamide tablets manufactured by Indian laboratories also failed the test [43]. Friability problems are related to the compression force or speed during manufacturing process, which are considered critical process parameters [44]. In addition, other studies detected pyrazinamide substandard formulations in India, Nigeria and Thailand due to mass variation test or because pyrazinamide was not successfully released during dissolution [37, 45, 46].

#### Ethambutol tablets

The packaging of all Mauritanian, taken from PNLTP and HP, and European ethambutol formulations studied was correct. Thus, their analyses were carried out obtaining the correct amount of declared value content; AV always below limit. All tested units of each drug product dissolved in less than 45 minutes releasing more than 80% of the declared drug content. As indicated in the above-mentioned publication, ethambutol tablets also failed the stated friability test [43]. Probably the reason for not complying with the friability test may be a failure in the manufacturing process, the hygroscopic of ethambutol or its excipient composition; the storage conditions and packaging are particularly important in this kind of product so as not to affect its quality attributes [47–49]. Nabirova et al (2017) identified also ethambutol batches from the same manufacturer laboratory that failed the physical inspection because they were chipped, dented or abrased [50].

#### Isoniazid tablets

The package of this isoniazid drug product sampled in CS IBN of Mauritania was in good conditions, although 2 dosage units were powdered during its handling before testing. Tablets had the amount of isoniazid labelled and contents were released above 80% at 45 minutes, complying with uniformity of dosage units and dissolution tests. However, these dosage forms did not fulfil friability test due to the loss of structure. Others isoniazid tablets manufactured in Asia did not meet with friability test or failed physical inspection, due to the poor conditions of some dosage units [43, 50]. High friability may lead to underdose of the patient and the emergence of MDR-TB.

#### Rifampicin and Isoniazid tablets

All rifampicin and isoniazid packaging of drug products taken from CDT, CS IBN and HP were in good conditions. The quality control of these 3 FDC tablets with the same batch was carried out according to pharmacopeial recommendations. In this case, every sample failed the CU test for 30 units, regardless of where they were collected. These results were due to the high variability of API content, which resulted in a high variability of standard deviation (SD) and an AV above the limit. This is in accordance with previously published studies from other African countries which also detected poor quality drugs of this APIs combination [9, 51, 52]. Regarding the dissolution test, as there is no specific monograph for this FDC tablets composition, the existing monograph for these APIs formulated in capsules was used to select the conditions of the test. RH-HP complied dissolution test for 6 tablets with % released greater than 80% and 85% for rifampicin and isoniazid respectively. However, RH-CDT and RH-IBN complied the test for 12 dosage units of each drug. Thus, all drug products studied complied with dissolution test, but with a high SD as observed in the CU test which does not comply.

#### Rifampicin, Isoniazid and Pyrazinamide tablets

1 European and 1 Mauritanian formulation from HP were analysed, and the poor conditions of the Mauritanian package was highlighted. For RHZ-CP, the content of each API was adequate in the case of the tablets analysed, and the AV was lower than 15 for all APIs. Furthermore, 12 FCT were needed for the dissolution test with a mean % released value of 88.1, 99.2 and 99.2 for rifampicin, isoniazid and pyrazinamide respectively and all units released more than 65%. In the case of RHZ-IBN, 30 FCT were analysed and this drug product did not meet the uniformity of dosage units test, because the AV of R was 15.1; higher than the limit established. Pouplin et al (2014) calculated the AV of this FDC tablets and found that the AV of the rifampicin and isoniazid were close to the limit value, although the formulation studied had complied with the content uniformity [53]. This drug product did not comply with the dissolution test because the % dissolved of rifampicin from 6 tablets was 29.1, with units below pharmacopeial limit of 50%, so no further tablets were tested. Ashokraj et al (2004) did an *in vitro* study with antiTB FDC tablets, and used 1 formulation from the same manufacturer laboratory. This formulation took longer to disintegrate than others and presented the most variable dissolution properties, which might explain the mean and high SD obtained in the present work. Furthermore, they found the formulations were stable under accelerated conditions during 1 month but showed higher variability in the results after their storage [54].

#### Rifampicin, Isoniazid, Pyrazinamide and Ethambutol tablets

Quality control was carried out on 2 different batches of the same Mauritanian drug product, which were collected in CDT, CS IBN and HP, and 1 European drug. In this case, packaging was correct. Each one complied with CU test, except RHZE-HP which exceeded the AV limit for ethambutol. In this case, the drug product quality could not be categorised because 20 more units would have to be analysed, and not enough tablets were available. Table 4 shows that the sampled drug products complied with dissolution test because 80% of all units dissolved. However, Kenyon et al (2001) detected substandard drug products of the 4 antiTB APIs first line treatment [51]. W Mweemba (2001) in this case detected an excess of ethambutol in FDC tablets which could lead to overdosing [52].

50% of 18 sampled drug products tested were categorized as substandard, 5 of them because they failed friability test, and the other 4 because AV obtained for uniformity of dosage unit test or its amount released from dosage form did not meet pharmacopeial limits. All UTs were manufactured by the same laboratory, and they failed the friability test, probably due to poor control of the manufacturing process. Non-compliance with this test may result in a dosage error because of loss during transport or handling of the tablets. Taking in account the sampling sites, the major substandard detection of drug products analysed completely, were from HP (2/2) and CS IBN (4/5).

It is well known that the AV determination must be performed to ensure the quality in terms of uniformity of the API content of every dosage form, as its statistical evaluation ensures batch compliance. In addition, the determination of AV has recently been evaluated to establish more restrictive values based on statistical analysis [55].

### Dissolution profiles comparison

The comparison of dissolution profiles would allow to assess batch to batch quality or to ensure continuity after Scale-Up and Post-Approval Changes. In this case, only levofloxacin 500 mg FCT was used, because it was the unique Mauritanian formulation with a European reference drug product, using their dissolution profiles to confirm their similarity. The European formulation, LVFX-DC, was used as reference drug for profiles comparison.

**Fig 2:**
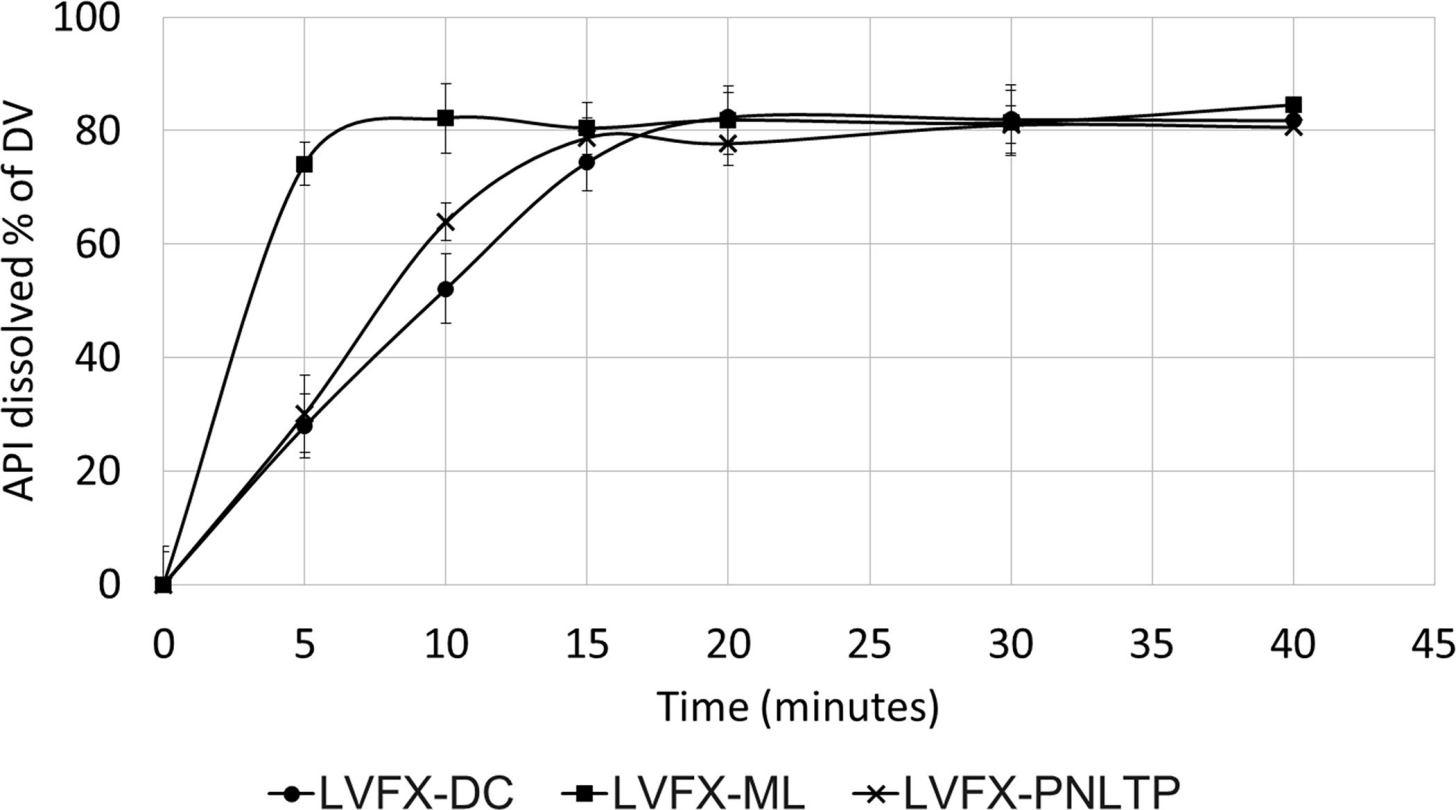
Dissolution profiles of levofloxacin drug products. LVFX-DC: Tavanic®. LVFX-ML: Levofloxacino Cinfa®. LVFX-PNLTP: Amesol®. API: Active pharmaceutical ingredient. DV: Declared value.

Dissolutions profiles of each drug product are shown in Fig 2. For LVFX-ML the asymptotic value was reached before 15 minutes and it was also the fastest to disintegrate, probably because it contains more disintegrants in its composition.

The similarity between LVFX-DC and LVFX-PNLTP was confirmed calculating *f1* and *f2* which were 8.4 and 55.9 respectively, establishing the pharmaceutical equivalence between these formulations.

### Stability studies

**Fig 3:**
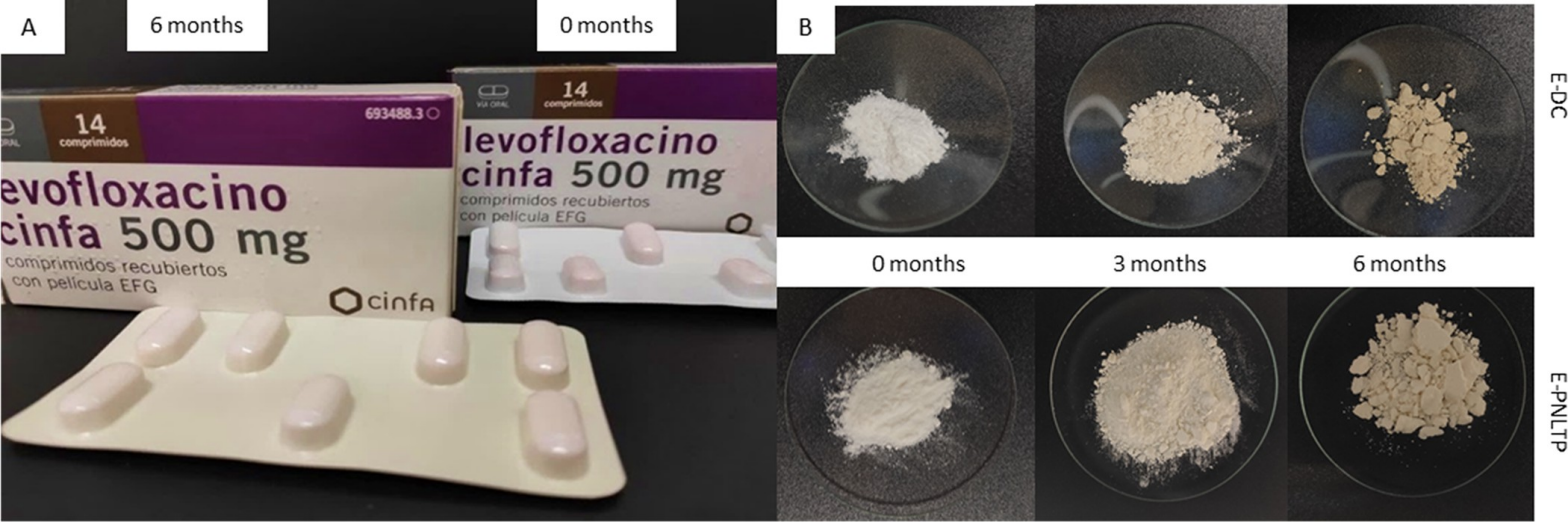
Appearance of packaging of LVFX-ML (A) and the solid powder ethambutol drug products (B) at different sampling times.

In order to study the influence of the storage conditions similar to Mauritanian climate zone the antiTB drug products that met the uniformity of dosage units and dissolution specification initially were selected and stored in a climatic chamber. At 3 months, these formulations were tested, carrying out pharmacopeial tests to check the compliance with specifications. The drug products which met requirements after 3 months were tested again at 6 months. Furthermore, the appearance of the primary packaging and dosage forms at time was observed, highlighting the changes observed as shown in Fig 3.

In this regard, drug products such as LVFX-ML packaging changed colour after 6 months in accelerated condition, but the tablets remained unchanged. The physical properties of E-DC and E-PNLTP tablets were also altered, due to wetting, forming agglomerates. In addition, the powdery solid became a darker yellow in appearance over time. Thus, ethambutol formulations can be affected by the high RH due to the hygroscopicity of this API. Packaging may not be suitable for the storage conditions, with 75% of RH as indicated by the ICH [36, 49] even for the Mauritanian drug product which only just complied.

Table 5 shows the results of uniformity of dosage units and dissolution test at 3 and 6 months of the drug products that complied with pharmacopeial tests performed at initial time. It is important to note that the drugs were stored in unsuitable conditions, according to the recommendations labelled on their packaging.

**Table 5:** Results of uniformity of dosage units and dissolution test during stability study. API: Active Pharmaceutical Ingredient.

		t = 3 meses						t = 6 meses					
		**UDU test**			**Dissolution test**		**Pass/ Fail**	**UDU test**			**Dissolution test**		**Pass/ Fail**
**CODE**	**API**	**% DV**	**AV**	**n**	**% released**	**n**		**% DV**	**AV**	**n**	**% released**	**n**	
LVFX-ML	LVFX	98.4 ± 1.0	2.6	10	94.3 ± 3.7	6	Pass	107.4 ± 1.2	8.8	10	98.6 ± 4.7	6	Pass
LVFX-DC		101.5 ± 0.5	1.4	10	94.4 ± 1.9	6	Pass	104.0 ± 0.4	3.5	10	97.0 ± 4.8	6	Pass
LVFX-PNLTP		97.9 ± 1.1	2.6	10	93.1 ± 3.6	6	Pass	98.4 ± 0.7	1.7	10	97.3 ± 3.4	6	Pass
Z-PNLTP	Z	101.0 ± 1.2	2.8	10	96.0 ± 1.5	6	Pass	102.5 ± 0.4	2.0	10	100.3 ± 0.6	6	Pass
E-DC	E	97.9 ± 0.9	2.7	10	70.9 ± 4.3	24	Fail						
E- PNLTP		95.1 ± 0.9	5.7	10	76.1 ± 7.4	12	Pass	92.0 ± 0.7	8.1	10	76.5 ± 7.5	24	Pass
RHZE-CDT	R	86.7 ± 4.3	22.1	30	89.5 ± 2.8	6	Fail						
	H	88.7 ± 3.0	17.1		96.6 ± 2.2								
	Z	103.4 ± 4.5	12.7		103.4 ± 1.4								
	E	97.2 ± 6.2	16.1		99.0 ± 1.3								

L: Levofloxacin; R: Rifampicin; H: Isoniazid; Z: Pyrazinamide; E: Ethambutol. ML: Manufacturer Laboratory. DC: Distribution company. PNLTP: Programme National de Lutte contre Tuberculosis et Paludisme. CDT: Center of diagnostic and treatment. company. UDU: Uniformity of dosage units DV: Declared value. AV: Acceptance value. n: Number of tablets tested.

Levofloxacin drug products complied with the all the pharmacopeial tests. The amount of E-DC dissolved at 45 minutes of 24 tablets decreased, failing dissolution test at 3 months because the API mean released was lower than 75%. Non-difference of quantity released was identified at 3 and 6 months for E-PNLTP, which met the dissolution test but close to the limit. For RHZE-CDT, CU test was carried out at 3 months, failing the test due to the high AV obtained. Furthermore, rifampicin and isoniazid content are near to the limit of Eur. Ph. for CU test. In this way, Ashokraj et al (2005) had previously demonstrated the degradation of rifampicin when stored under accelerated conditions [7].

**Fig 4:**
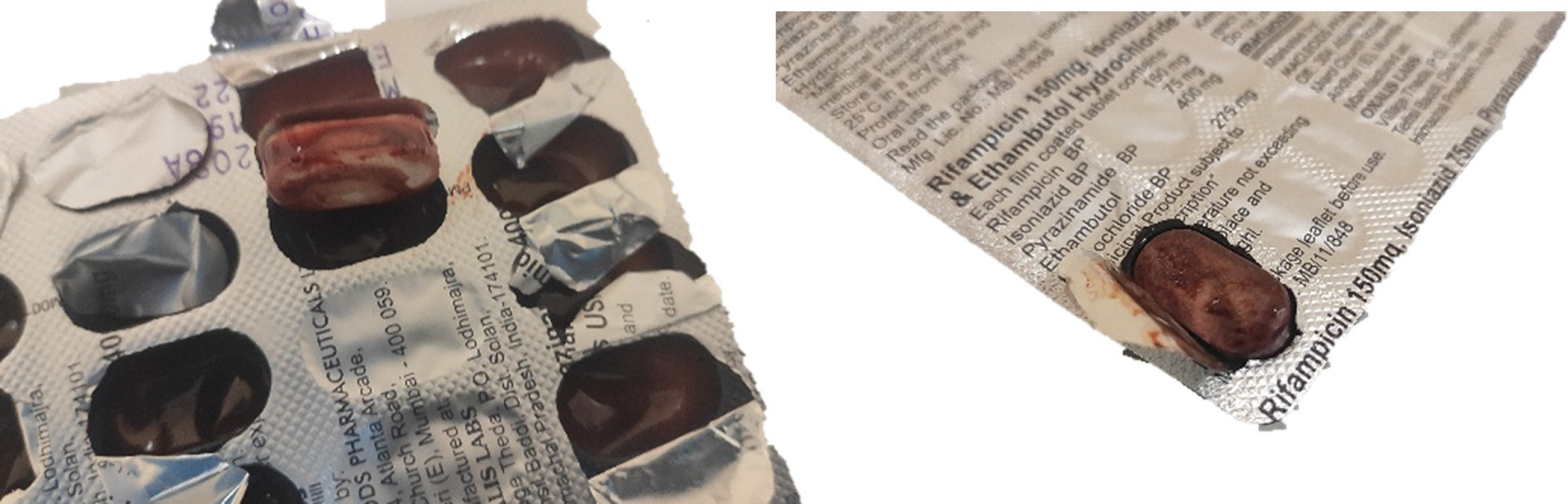
Appearance of two units wetted of RHZE-CDT at 3 months.

Packaging and deteriorated pharmaceutical forms from RHZE-CDT at 3 months of storage are shown in Fig 4. Its blister seemed to be in good conditions, but two units were wet, and had lost their coating film, and following analysis degradation was higher than 40% for rifampicin, isoniazid and ethambutol.

Hydrazine content of 3 tablets from RHZE-CDT was analysed, without detecting this impurity although formation may be major under accelerated conditions.

## Conclusion

All European and 3 Mauritanian formulations of the 18 drug products studied, complied with the pharmacopeial test after collection, meaning that 9 drugs of the 13 Mauritanian samples were categorized as substandard and 1 could not be determined. The 3 remaining formulations met specifications. Mauritanian substandard drugs failed friability test for every UT, CU test for 4 FDC drugs due to the high AVs obtained or dissolution test because the content of rifampicin was not released. Furthermore, hydrazine was not detected in any sample even during the stability tests.

Unsuitable storage, because of shipping, storage conditions, or packaging are unable to ensure the quality of medical products throughout the supply chain and may be the cause for the substandard products. The accelerated conditions mainly influenced the dissolution rate of ethambutol [49] and the uniformity of the content of the FDC tablets. However, the storage conditions were not affected in the case of levofloxacin or pyrazinamide drugs.

Quality assurance of medical products is not only achieved through quality control of these products, but also requires regulatory measures by governments. These measures would establish safe and reliable pathways to ensure the quality of these products from manufacture to dispensing. In this particular case, the reduction of the substandard products could be achieved by improving storage conditions during distribution and dispensing centres.
